# Framing Undergraduate Public Health Education as Liberal Education: Who Are We Training Our Students To Be and How Do We Do That?

**DOI:** 10.3389/fpubh.2017.00009

**Published:** 2017-02-10

**Authors:** Marc T. Kiviniemi, Sara L. C. Mackenzie

**Affiliations:** ^1^Department of Community Health and Health Behavior, University at Buffalo, SUNY, Buffalo, NY, USA; ^2^Department of Health Services, University of Washington, Seattle, WA, USA

**Keywords:** undergraduate public health education, liberal education, professional preparation, program design, curriculum design

## Abstract

The rapid development of the undergraduate major in public health over the past 15 years has led to a debate about the most appropriate framing for the degree. Should it be viewed as a liberal education degree (akin to academic disciplines such as psychology and political science) or as a professional training degree (akin to disciplines such as nursing and management)? This paper presents an overview of both the liberal education and the professional training degree approaches to the undergraduate public health degree. The reality of public health work in the modern era and the constraints on undergraduate-level training lead to our conclusion that the liberal education framing is a more optimal way to design the degree program. Such a framework optimizes career opportunities, especially long-term opportunities, for graduates, acknowledges the reality of the complex and diverse career paths that one can take under the general umbrella of public health, and accounts for the important role of critical thinking skills in undergraduate education. Ultimately, the distinction between liberal education and professional training may be fuzzier than the debate often highlights—an intentional, well-designed, and thoughtfully implemented undergraduate public health curriculum can address the range of student needs underlying both the liberal education and professional training approaches to the degree, thus optimizing both learning goals and career outcomes for undergraduate public health students.

## Introduction

The landscape of public health education has changed dramatically in recent years. Over the past 15 years, we have seen a rapid expansion in public health education for undergraduates in the United States. In 1992, 759 undergraduates received public health degrees in the United States; by 2014, this had increased to 9,661 (1, 2). Undergraduate public health degree programs are developing within Schools of Public Health, programs of Public Health, and 4- and 2-year institutions without graduate programs in Public Health [for an overview of the history and growth of the undergraduate major, see Ref. (3)].

Developing new programs (as well as substantively revising existing programs) provides a unique opportunity to develop best practices for curriculum design. Accreditation requirements for undergraduate public health programs have been developed (4), and suggested learning outcomes have been articulated (5, 6). While the development of the learning outcome domains and accreditation criteria are useful, they do not clearly answer two critical questions necessary to optimally develop and deliver effective public health programs. First, we must ask—“who are we training our students to be?” That is, what are the range of next steps we are preparing students for in terms of employment and further study and what abilities do students need to succeed in that range of next steps? Once we have answered the first question, the second question becomes—what guiding philosophy for curriculum development best achieves our goal of preparing students with the abilities necessary to succeed at their next steps after graduation.

Both these questions are critical to addressing an ongoing debate in the world of undergraduate public health education—whether undergraduate public health training should be framed within a liberal arts framework versus centering on a professional public health framework (3). Some argue that the degree should be specifically designed for workforce preparation and that it is best conceived as a professional bachelor’s degree (3, 7), while others argue that at the undergraduate level, public health or population health should be framed in the context of liberal education [(8, 9); for a discussion of the various framings of the degree, see Ref. (10)].

This is not a purely philosophical debate—program design decisions have implications for curricular focus, academic advising, faculty mentoring, career counseling, and the expectations of students, faculty, and the workforce. A liberal education focus in contrast to a professional focus will influence post-graduate employment options and careers for students and, more importantly perhaps, the ability of population health-educated citizens to bring that lens to a range of careers. In addition, the design choices have significant implications for articulations between 2- and 4-year undergraduate public health degrees and graduate public health education. This debate is not new and echoes a broader debate about the purpose and structure of undergraduate education across higher education. However, given that many public health faculty and practitioners are newly engaging with undergraduate education, we feel it is important to dig deeper into the questions of who are we training our graduates to be and how that impacts curriculum design.

## Who are We Training Our Students to be?

Higher education training in public health is providing students with skills to interface in some capacity to improve the health of populations. If we start with the end point in mind, we need to consider what the nature of the public health is. The pressing public health problems faced locally and globally rapidly change. In the 2–3 years preceding the writing of this paper, the public health community faced the emergence of a pandemic of Ebola virus, a restructuring of insurance policies under the Affordable Care Act of 2009, emergence of the relatively novel mosquito-borne Zika virus, a community-wide clean water crisis caused by mismanagement of a public works infrastructure, gun violence, and an opioid epidemic. The nature of public health and the regular emergence of new challenges require not only a firm understanding of the *principles* of public health but also the ability to apply those principles flexibly to new challenges that might have unique characteristics and unique needs for solutions.

The increased recognition of the role of social determinants, including education, housing, employment, and urban design, has expanded the range of people who do public health work beyond those traditionally recognized as public health professionals (11). Current discussions call for a revisioning of Public Health to an upgraded 3.0 version that is “a modern version that emphasizes cross-sector collaboration and environmental, policy, and systems-level actions that directly affect the social determinants of health” (12). The rapid expansion of undergraduates receiving public health education and a need for an expanded spectrum of people working to improve the health of populations means that bachelor-level public health trained students have the opportunity to move into a broad range of career options to influence the health of their communities.

In addition, we are training undergraduates not only for their first job but also, ideally, for a career that will likely span at least three decades. Given that public health issues, techniques, and domains change at a much quicker pace than the average person’s career, a focus on teaching analytical, thinking, and communication skills provides a flexible skill set that enables the students to change and adapt as the public health challenges and related job opportunities evolve over time.

If we hope to perform an upgrade to Public Health 3.0 (12), we will need a future where graduates of public health programs enter careers representing a cross-section of society—health professions, business, law, urban planning, advocacy, politics, and education—and take population health frameworks into all professions to truly address social and environmental determinants of health. Thus, undergraduate public health curriculum design must ensure that we prepare students for a broad range of roles and types of jobs. What are the educational framing options we can consider?

## What do We Mean When We Say Liberal Arts/Liberal Education Framing?

Liberal arts is a term that is used in many ways and often misinterpreted in just as many. For some, the liberal arts denotes a “traditional” set of disciplines such as literature, history, and philosophy. For others, the term liberal triggers an inaccurate association with a particular political orientation toward addressing problems—the educational meaning has never been intentionally linked to a political orientation. Finally, given the labeling at many institutions, liberal arts also sometimes means a particular decanal unit within the university’s organizational structure.

Given this mix of labels, it is useful to draw a distinction between the terms liberal arts and liberal education and use the term liberal arts for the “traditional” disciplines (such as history, philosophy, and literature). Liberal education, the framework around which we will focus our discussion of undergraduate public health, is then allowed to have its own distinct meaning (13).

Liberal education is an approach that is independent of any specific disciplinary boundaries. The hallmarks of a liberal education approach are that the primary learning outcomes center on acquiring transferable skills to obtain, evaluate, analyze, and apply information to understand and address problems. The potential learning outcomes associated with such an overarching approach are numerous [for some examples, see Ref. (13), p. 22]. At its core, the focus on critical thinking skills and using knowledge to address and solve problems is perhaps the key defining feature (14). Others have noted that a focus on “real world” issues, developing the ability to see a problem from multiple perspectives, to use more than one disciplinary lens to look at the problem, and a focus on communication skills also highlight the liberal education approach (15, 16). Finally, a recent articulation of essential learning outcomes that a liberal education should strive to achieve seeks to combine these foci, arguing that liberal education should provide knowledge of human cultures and the physical and natural world; intellectual and practical skills (including but not limited to critical and creative thinking, quantitative literacy, and teamwork and problem solving); personal and social responsibility (including civic engagement, intercultural knowledge, and ethical reasoning); and integrative and applied learning (17).

One class of definitions of liberal education with which we, for our purposes in undergraduate public health, vehemently disagree is the characterization that liberal education centers on learning for learning’s sake (18). We believe and hope to show that a liberal education approach to the undergraduate public health curriculum prepares students for a breadth of employment options in public health and for broader roles as educated citizens making decisions about population health issues in a complex, modern world. As a framework, public health liberal education is in sync with public health’s central mission of actually improving population health and well-being and thus not just learning for learning’s sake.

### Liberal Education and Public Health

What does a liberal education approach to public health look like? One answer is provided by the Undergraduate Public Health Learning Outcomes, released by the ASPPH in 2011 (19). These outcomes were inspired by the Institute of Medicine’s recommendation for an Educated Citizenry (20). Importantly for framing the public health degree in a liberal education context, the Undergraduate Public Health Learning Outcomes draw from and mesh with the AACU’s articulation of essential learning outcomes for a liberal education degree (see http://aacu.org/leap for details) and directly apply those broader earning objectives to the public health context.

In these outcomes, the defining characteristic of the content focus becomes the relationship of content to individual and population health. This includes the intersection of the physical/biological and the human/cultural/environmental to determine health; the necessity of gathering information, reasoning about that information, and using it to solve problems in complex and team-oriented ways; the focus on not just acquiring knowledge but using it in ways that are effective, culturally appropriate, ethical, and innovative to solve public health problems; and to integrate and apply knowledge from a diversity of disciplines within and outside of public health to solve problems.

The use of public health as a context for liberal education is not wholly novel. Fraser (21), in a now classic article on epidemiology as a liberal art, argued that the public health discipline of epidemiology has many of the core features of the liberal arts (his term—given the distinction described above, we would say liberal education). Fraser argues that epidemiology involves acquiring and using a set of habits of thought and critical thinking skills (the scientific method) to approach problems; that it involves applying reasoning skills of a variety of types to take observations (“people who ate at restaurant A got the virus and people who ate at restaurant B didn’t”) and draw conclusions from them. In addition, it involves taking knowledge gathered and reasoned upon and using that knowledge to determine ways of addressing problems effectively (21).

## What do We Mean When We Say Professional Education?

In contrast to liberal education, professional education is a systematic, workforce-oriented, and discipline-specific approach to training. In contrast to liberal education’s focus on meta-level thinking, reasoning, and action skills contextualized within the framework of a particular disciplinary/professional body of knowledge, professional education centers around preparing students with a given set of skills and concrete domain knowledge necessary to enter a defined role or profession. Within the domain of undergraduate education, undergraduate programs in nursing, engineering, accounting, and teacher education are typically conceptualized as professional education programs. Closer to the public health domain, environmental health programs (accredited for professional training since 1967 by the National Accreditation Council for Environmental Health Curricula),^1^ health informatics/health education (since 1942),^2^ and dietetics programs (Academy of Nutrition and Dietetics)^3^ are all examples of typically professionally oriented undergraduate programs.

In public health, the masters in public health has long been conceptualized as a professional training degree, preparing students coming directly from undergraduate studies and individuals with advanced training in another health field (e.g., MD/MPH) for professional roles in traditional public health fields.

At the undergraduate level, professional baccalaureate programs have focused requirements based on specific workforce skills and generally include significant levels of required field experience in the form of internships or practica (for example, requirements ranging from 180 h for Environmental Health to 1,200 h in Dietetics). With a set number of credits associated with a baccalaureate degree, programs with more focused discipline-specific professional competency development reduce the opportunities for breadth of competency development—specialization versus transferable skill development.

If we argue that the general public health baccalaureate programs are professional programs, surveying workforce for necessary skills is critical. A systematic workforce-oriented approach requires consideration of the specific workforce or profession we are training students to enter (22). The traditional public health workforce would fulfill the core functions or essential functions of public health including assessment, policy development, and assurance with the public health system including an extensive network of public and private agencies and organizations (23). However, the expanded range of career options that influence the health of communities discussed earlier means that identifying and targeting clear professional skills at the undergraduate level is complicated and perhaps requires a narrowing of focus too soon in an undergraduate’s education to truly meet the needs of a new public health workforce.

## Moving Forward

So where does this analysis leave us? What practical take aways should those running undergraduate public health programs or developing future undergraduate public health programs take from our analysis of the liberal arts and professional education perspectives? At the most basic level, we hope that this analysis of the implications of these two guiding frameworks highlights the critical need to *be intentional* in selecting a framework, developing desired learning outcomes, and shaping a curriculum based on those decisions. These decisions about framework go well beyond simply shaping the curriculum—they shape how we encourage faculty to design and teach courses, how we advise students about the various career paths available to them, and how we talk with external constituencies and with parents of students about the value of the public health major.

Having said that, we also strongly believe that the liberal education perspective has several advantages in meeting the needs of undergraduate public health students and of the broader public health community. We strongly believe that framing the undergraduate public health degree as a liberal education degree offers substantial benefits for students and the health of the public. The focus of liberal education as “a philosophy of education that empowers individuals, liberates the mind…, and cultivates social responsibility. Characterized by challenging encounters with important issues, and more a way of studying than specific content ….” [(13), p. 25] will best prepare students to address the future health needs of our communities. The future of public health work inherently calls for a wide range of people trained in population health concepts and able to be flexible, adaptable problem solvers with an ability to apply and modify principles as needed to new contexts and new challenges. The liberal education approach to undergraduate training is, at its core, an approach designed to meet these sorts of challenges.

Although we take the position that if one is forced to choose a perspective, one should choose liberal education, we would also point out that careful consideration of the two frameworks suggests that liberal education versus professional degree framings may be a false dichotomy—in fact, there may be substantial middle ground where the two can meet. Meeting in this middle ground, we think, requires a mindset shift on the part of both those in the liberal education and the professional training camps.

For those in the liberal education tradition, we would argue that it is useful to keep in mind that although the core characteristics of liberal education center on higher order thinking skills, those skills are learned and applied in a specific disciplinary context. When designing a liberal education curriculum, there is nothing to stop the educator from selecting disciplinary material with an eye toward initial professional roles—teaching problem solving skills using problems curated to match those that will be encountered in initial professional positions, addressing critical thinking by selecting information and arguments relevant to job roles, and establishing internship opportunities in targeted areas for students planning direct workforce entry provides the potential to maintain a core focus on the higher order liberal education skills while still enhancing job transfer and professional applicability of the public health education.

For those who start in the professional training camp, we would argue that focusing thinking on those job-readiness skills that are common across the range of public health career outcomes can draw in a stronger liberal education perspective. The breadth of bachelor’s level public health jobs makes it challenging (if not impossible) to fully train graduates for each of the multitude of possible career outcomes. However, we would argue that many of the skills that are a focus of liberal education are common across many if not all career paths. AACU has made the point that “a liberal education is a practical education because it develops just those capacities needed by every thinking adult: analytical skills, effective communication, practical intelligence, ethical judgment, and social responsibility” [(13), p. 26]. To put it in professional degree terms, the liberal education approach seeks to equip students with a set of skills enabling them to gather information, synthesize, and analyze that information to form judgments, implement action based on those judgments, and to ensure that each of those steps is done with a focus on professional and personal ethics and the needs of the broader society. This “job readiness” criteria for the health professions was recently articulated in a report on the future of health professional education [(24), p. 1,924]—“all health professionals in all countries should be educated to mobilize knowledge and to engage in critical reasoning and ethical conduct so that they are competent to participate in patient and population-centered health systems as members of locally responsive and globally connected teams.”

This focus on liberal education skills as a route to job readiness is, we believe, the best way to meet the needs of the diverse range and growing number of undergraduate public health students. Rounding out the “middle ground” between the liberal education and professional training camps, though, requires considering situations where a student in a liberal education program may be best served by a focus on professional training components. For those students focused solely on obtaining bachelor’s level employment in public health, we would argue that early-stage academic advising and career counseling should be provided to aid the student in narrowing the broad range of possible job outcomes. Doing so would then allow the student to, with advising guidance, select coursework and experiential learning opportunities that would provide practical, professional training for that more constrained career path.

## Conclusion

Criteria developed by the ASPPH to guide program development at the undergraduate level are inherently flexible, given that programs have the ability to apply a framework that best meets the needs of their students. The liberal education framing, we would argue, provides the best of both worlds. Public Health as liberal education is job training—it is training for a wider range of jobs and careers by teaching students transferable skills using population health as the context and value system. Engaging students, staff, and faculty in discussions around the breadth of jobs and career pathways open to graduates is important for career counseling and advising. Students should understand how to apply the skills they have learned to a range of career options. Programs can be more intentional about developing internships, partnerships, and opportunities for students in a more expansive, cross-sector manner that best serves our communities.

## Author Contributions

MK and SM contributed to conceptualization, writing, and editing of this manuscript.

## Conflict of Interest Statement

The authors declare that the research was conducted in the absence of any commercial or financial relationships that could be construed as a potential conflict of interest.

## Footnotes

^1^http://ehacoffice.org/accred-guide/under-guide.php.

^2^http://www.cahiim.org/about%20us/history.html.

^3^http://www.eatrightacend.org/ACEND/content.aspx?id=6442485421.
